# Immune-related LncRNAs scores predicts chemotherapeutic responses and prognosis in cervical cancer patients

**DOI:** 10.1007/s12672-024-00979-1

**Published:** 2024-04-14

**Authors:** Weijie Tian, Songsong Tan, Jun Wang, Ping Shen, Qingfen Qin, Dan Zi

**Affiliations:** Department of Gynecology, Guizhou Provincial People’s Hospital, Medical College of Guizhou University, Guiyang, Guizhou People’s Republic of China

**Keywords:** Cervical squamous cell cancer, Immune, Long non-coding RNA, Prognosis, Chemotherapeutic Responses

## Abstract

**Background:**

Long non-coding RNAs (LncRNAs) regulating the immune microenvironment of cancer is a hot spot. But little is known about the influence of the immune-related lncRNA (IRlncRs) on the chemotherapeutic responses and prognosis of cervical cancer (CC) patients. The purpose of the study was to identify an immune-related lncRNAs (IRlncRs)-based model for the prospective prediction of clinical outcomes in CC patients.

**Methods:**

CC patients’ relevant data was acquired from The Cancer Genome Atlas (TCGA). Correlation analysis and Cox regression analyses were applied. A risk score formula was formulated. Prognostic factors were combined into a nomogram, while sensitivity for chemotherapy drugs was analyzed using the OncoPredict algorithm.

**Results:**

Eight optimal IRlncRs(ATP2A1-AS1, LINC01943, AL158166.1, LINC00963, AC009065.8, LIPE-AS1, AC105277.1, AC098613.1.) were incorporated in the IRlncRs model. The overall survival (OS) of the high-risk group of the model was inferior to those in the low-risk group. Further analysis demonstrated this eight-IRlncRs model as a useful prognostic marker. The Nomogram had a concordance index of survival prediction of 0.763(95% CI 0.746–0.780) and more robust predictive accuracy. Furthermore, patients in the low-risk group were found to be more sensitive to chemotherapy, including Paclitaxel, Rapamycin, Epirubicin, Vincristine, Docetaxel and Vinorelbine.

**Conclusions:**

An eight-IRlncRs-based prediction model was identified that has the potential to be an important tool to predict chemotherapeutic responses and prognosis for CC patients.

**Supplementary Information:**

The online version contains supplementary material available at 10.1007/s12672-024-00979-1.

## Introduction

Cervical cancer (CC) is still the common cause of disease-related mortalities in women, leading to nearly 300,000 deaths worldwide [1]. Cervical squamous cell carcinoma (CSCC) is the primary pathological subtype and comprises the most CC cases [2]. Despite the utilization of treatment modalities such as surgery, radiotherapy, and chemotherapy, these therapies have shown limited efficacy in patients with advanced-stage disease [3–5]. The International Federation of Gynaecology and Obstetrics (FIGO) stage is the primary prognostic indicator for CC. However, the FIGO stage cannot differentiate the various heterogeneity of CC in terms of clinical behavior. Patients with the same FIGO stage may often present obviously different clinical outcomes. Therefore, identifying new prognostic indicators reflecting the heterogeneity of CC is essential and can facilitate individualized treatments for patients with an otherwise poor prognosis.

Increasing evidence showed that immune system disruption might lead to tumour progression and metastasis [6, 7]. Malignant cells can escape immunosurveillance by reducing the expression of major histocompatibility complex class I molecules. Cervical adenocarcinoma has impaired recruitment of CDC1 and CD8 + T cells [8, 9]. Higher CCL22 + cell infiltration is negatively associated with prognosis in CC patients [9]. LINC00240 promotes natural killer T cell cytotoxic activity in CC and enhanced the growth, invasion, and migration of CC cells [10].

LncRNAs are a class of non-coding RNAs (ncRNAs) with longer than 200 nucleotides. With the development of transcriptome sequencing, it is clear that over 70% of the genome is transcribed into RNA, and the majority of them are ncRNAs [11]. lncRNAs are involved in various transcriptional and post-transcriptional gene regulatory processes and play crucial roles in the tumour immune response, including immune recognition and immune infiltration [11]. Numerous tumor-associated lncRNAs have been recognized as tumor cell factors that regulate tumor cell escape of immunosurveillance. These immune-related lncRNAs (IRlncRs) may play essential parts in immunotherapy resistance and further impact the prognosis of cancer patients [12]. Specifically, a subset of lncRNAs act as immune-related lncRNAs (IRlncRs) by regulating immune responses in the tumor microenvironment. This subset of lncRNAs has been shown to play a key role in modulating tumor immunosurveillance, immune cell infiltration into the tumor microenvironment, and sensitivity of cancer cells to immunotherapy treatment [12, 13]. Cao et al. identified an immune-related five-lncRNAs signature positively correlated with tumour immune cell infiltration and the poor prognosis in bladder cancer patients. The association of IRlncRs expression and the clinical outcomes of CC patients is reported, but the results lack validation [12].

The development of new prognostic markers is essential considering the inherent heterogeneity of cervical cancer, guiding personalized treatment strategies. Reliable assessment of chemotherapy responses and determination of prognostic risk would enable physicians to tailor more accurate treatment plans to improve outcomes. The IRlncRs signature holds strong potential in risk stratification and chemotherapy selection for cervical cancer patients. By evaluating prognosis and chemotherapy sensitivity, the IRlncRs model can provide a basis for clinical decision-making, offering patients the most likely successful treatment strategies based on their molecular risk profiles. To achieve this, we developed an innovative IRlncRs model and performed preliminary in vitro validation, demonstrating its ability to distinguish high-risk and low-risk cervical cancer patients with significant differences in overall survival. Further analysis confirmed the prognostic predictive capability of this model. Additionally, this model demonstrated utility in predicting chemotherapy response, with high-risk patients showing resistance to several commonly used drugs.

## Materials and methods

### Patient datasets

CC transcriptome RNA-seq data in the format of Fragments Per Kilobase Million and the corresponding clinical data of The Cancer Genome Atlas (TCGA) (GDC, https://gdc.cancer.gov/)were downloaded.

### Data preprocessing and normalization

The raw RNA-sequencing count data from TCGA cervical cancer cohorts was preprocessed to ensure normalization and integrity for downstream analyses. Briefly, quality control was first performed using FastQC to assess attributes like guanine-cytosine content, overrepresented sequences, and duplication levels. Reads were then trimmed and filtered to remove adapters and low-quality bases using Trimmomatic.

The RNA-seq pre-processed data was quantified and normalized using the Trinity pipeline (https://github.com/NCIP/Trinity_CTAT) to generate normalized gene-level count data. This pipeline maps reads, assembles transcripts, estimates abundances, and extracts differentially expressed features. Normalization was conducted using the trimmed mean of M-values (TMM) method to account for differences in sequencing depth between samples using the EdgeR R/Bioconductor package. TMM normalization controls for library size variability via scaling based on the ratio of read counts between samples. Normalized expression data is represented as counts per million (CPM).

Samples with > 50% missing lncRNA expression data were excluded from analysis to avoid technical bias. For the remaining samples, missing values were imputed using a k-nearest neighbor algorithm with k = 10 neighbors. Imputation was conducted using the Bioconductor impute package. This allowed retention of samples with some missing data rather than complete exclusion.

Additional filtering of lncRNAs was conducted to restrict analysis to those with evidence of abundance and variation across samples. LncRNAs expressed at ≥ 0.5 CPM in at least 10% of samples and with an interquartile range greater than 0 were retained.

### LncRNA profile mining

Three gene sets, “immune response(M19817)”, “immune system development(M3457)”, and “immune system process(M13664)”, were acquired from the Molecular Signatures Database. LncRNAs with abundance lower than 0.5 and lncRNAs of normal tissues were excluded. Then, immune-related genes were acquired from the above three gene sets. The Pearson correlation test analyses the correlation between the immune-related genes and lncRNAs. Absolute value of correlation coefficient > 0.5 and p < 0.001 were defined as IRlncRs.

### Real‑time quantitative PCR

Total RNA from cell lines (Hela cell and HCerEpiC cell) was isolated using Trizol reagent (Invitrogen, USA) according to the manufacturer’s instructions. cDNA Synthesis Kit (TaKaRa, Japan) was utilized to generate cDNA. 4.5 μL diluted cDNA (1:50) was used as the template in a 10 μL qPCR reaction using the ABI 7500 fast real-time PCR system (Applied Biosystems). GAPDH was used as a reference. The relative expression level was calculated by the 2^−ΔCt^ method. Table 1 shows the sequences of the forward and reverse primers of eight examined IRlncRs (ATP2A1-AS1, LINC01943, AL158166.1, LINC00963, AC009065.8, LIPE-AS1, AC105277.1, AC098613.1.).

**Table 1:** The forward and reverse primer sequences of eight examined IRlncRs (ATP2A1-AS1, LINC01943, AL158166.1, LINC00963, AC009065.8, LIPE-AS1, AC105277.1, AC098613.1.) for performing real-time PCR assay

Gene symbol	Primer	Primer Sequence (5′-3′)
ATP2A1-AS1	Primer_F	GAGGAGAATCCGCACCAGGA
	Primer_R	TAGCCACAAAGTCTTGGGTGT
LINC01943	Primer-F	CAGGAAGCGTGAGGACAGAA
	Primer-R	AACCAGACTGATGCCACAGG
AL158166.1	Primer-F	TGAGCATAGCCTCCACTCCT
	Primer-R	AGACAGCACTGTCAGTCACG
LINC00963	Primer-F	GAACTGCCTTTGGAAGCAAG
	Primer-R	AGGAGTTCGAGGCTGCAGTA
AC009065.8	Primer-F	TTAGCTGGGCTGCGTTTACA
	Primer-R	CCACTCTCCCACCTCCCTTA
LIPE-AS1	Primer-F	CTCTGTCTCCGCCCCCTAAT
	Primer-R	TTCTCAAGCATGCGTCGTTC
AC105277.1	Primer-F	GTGACCAGGTACTGGGGAAA
	Primer-R	AATGAGGTTCCACACCTGCT
AC098613.1	Primer-F	GGGGAAAATCATCTCCCATT
	Primer-R	TCACATTGCTCTGCCTCATC

### Model development

Univariate and multivariate Cox regression models were utilized to identify lncRNAs prognostic of overall survival and build a predictive risk score formula. The Cox proportional hazards regression model was selected because it allows assessment of the association between continuous gene expression data and censored survival outcomes while adjusting for the effects of other covariates. Univariate Cox regression was carried out to extract IRlncRs correlated with the OS of patients with CC at p < 0.05. Next, only the IRlncRs with a statistical significance of p < 0.01 were further enrolled in the stepwise multivariate Cox regression analysis to extract optimal IRlncRs independently associated with prognosis at p < 0.05. Finally, the survival‑related IRlncRs(sIRlncRs) model (risk score) was constructed according to the regression coefficients with lncRNA expression. In other words, the prognostic risk score was formulated based on a linear combination of the expression level of theses IRlncRs multiplied by the regression coefficients derived from the multivariate Cox regression analysis, as mentioned above [14]. Patients were grouped into a high- and low-risk group according to the median value of risk scores.

Kaplan–Meier analysis was employed to validate survival differences between the high-risk and low-risk groups stratified by the risk score formula. This non-parametric analysis was chosen because it is well-suited for estimating group survival functions over time while accounting for censoring, which was essential for the overall survival endpoint that had censored observations (patients still alive at last follow-up). The Kaplan–Meier estimator also provides median survival times and key quantified survival statistics for each risk group. Using this method enabled validation of the risk score by verifying poorer survival prognosis in the high-risk group compared to the low-risk group in a time-to-event analysis context.

### Independent prognostic analysis

We applied both single and multifactorial analyses to validate the validity of the risk score being an independent prognostic marker for CC. The receiver operator characteristic (ROC) curve was utilized to assess whether the risk score’s predictive power was reliable. The relationship between clinical traits and sIRlncRs was also studied. We also employed the PCA (principal components analysis) method to demonstrate the distribution patterns between the low- and high-risk groups. GSEA was applied to explore the distinct functional phenotypes between the high-risk and low-risk groups.

### Construction a predictive nomogram

A prognostic nomogram including risk scores and clinical features for predicting the likelihood of 3-, and 5-year OS was developed by R “rms” package. The calibration curves and C-index were used to evaluate the predictive accuracy of the nomogram [15].

### Prediction of chemotherapeutic response

The clinical response of each CC patient in high- and low-risk groups to chemotherapy was estimated based on the Genomics of Drug Sensitivity in Cancer (GDSC; https://www.cancerrxgene.org/) data. Twenty commonly used chemotherapy drugs of CC, were selected for the chemotherapeutic response prediction through the ridge regression using the “OncoPredict” R package [16]. The half-maximal inhibitory concentration (IC50) predicted of each CC patient was used to assess differential chemotherapeutic response.

### Statistical analysis

Statistical analyses were carried out by the R statistical programming environment (version 4.0.2). Correlations between the immune-related genes and lncRNAs were tested using the Pearson correlation test. Sensitivity and specificity of signature were determined by ROC curves representing its power to differentiate the different groups. The R package “survivalROC” was used to calculate the area under the curve [17], and the “survival” R package was loaded to figure survival analysis [18].

## Results

### The analysis process of this study

**Fig. 1:**
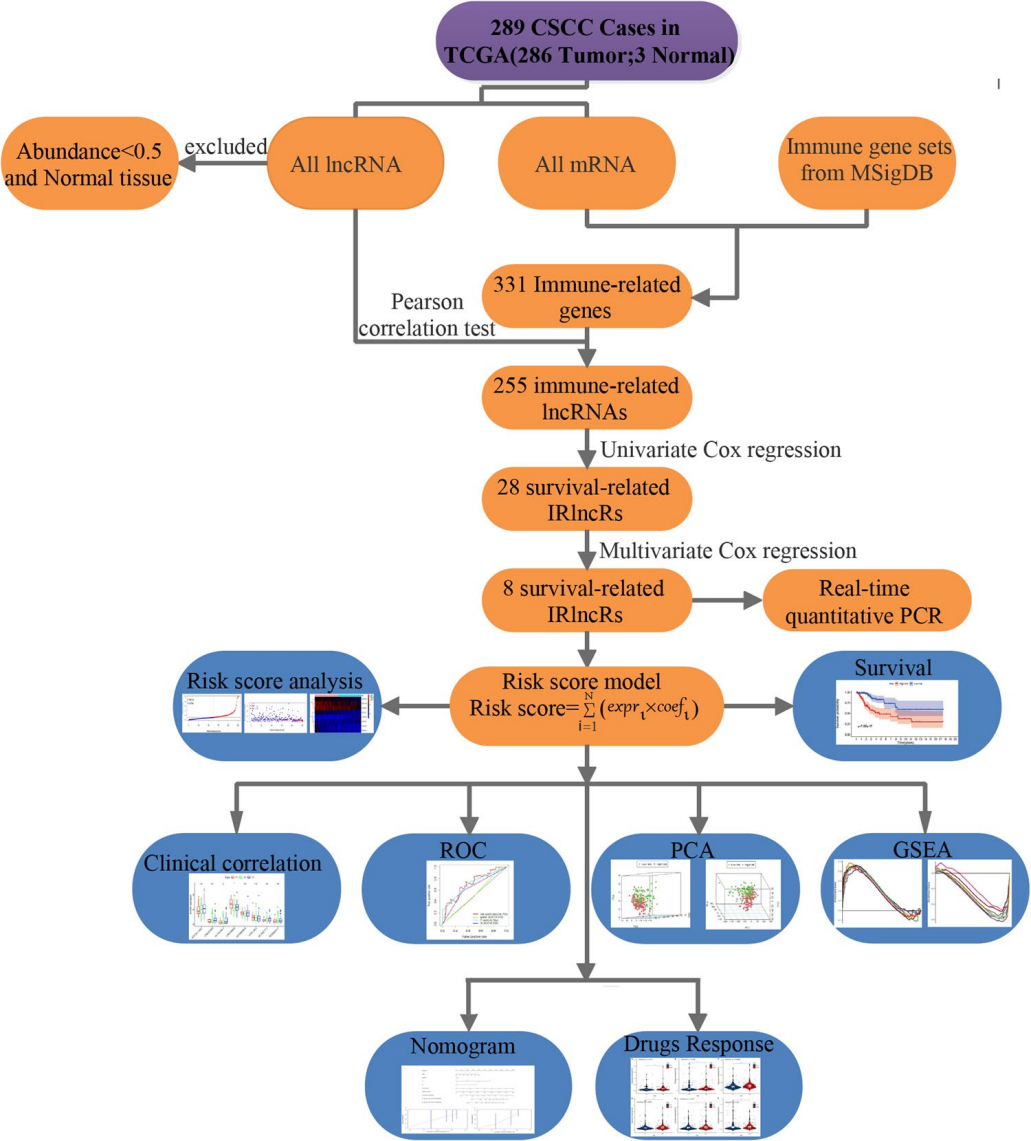
Analysis of the workflow of this study

Figure 1 displays the analysis process of our study. We downloaded transcriptome RNA-seq data and corresponding clinical data of 289 cases of CC from the TCGA database. Among these cases, there were 253 CSCC patients, 33 cervical adenocarcinoma patients, and 3 healthy control patients (Additional file 3: Table S1). Then, the RNA-seq data were divided into mRNA and lncRNAs data. LncRNAs with an abundance less than 0.5 and normal tissue lncRNAs were excluded. We identified 331 immune-related genes from gene sets of MSigDB, of which 255 lncRNAs were IRlncRs validated by correlation analysis (Additional file 1: Fig. S1). Next, we identified 28 IRlncRs that were associated with the prognosis of CC. We further optimized theses IRlncRs by stepwise multivariate Cox regression, and eight sIRlncRs were utlized to formulate the risk score model. Finally, we utilized the risk score model for a series of subsequent analyses, including survival analysis, risk score analysis, clinicopathological characteristics, ROC curve analysis, PCA, and GSEA.

### Construction of an IRlncRs-based risk score model

**Fig. 2:**
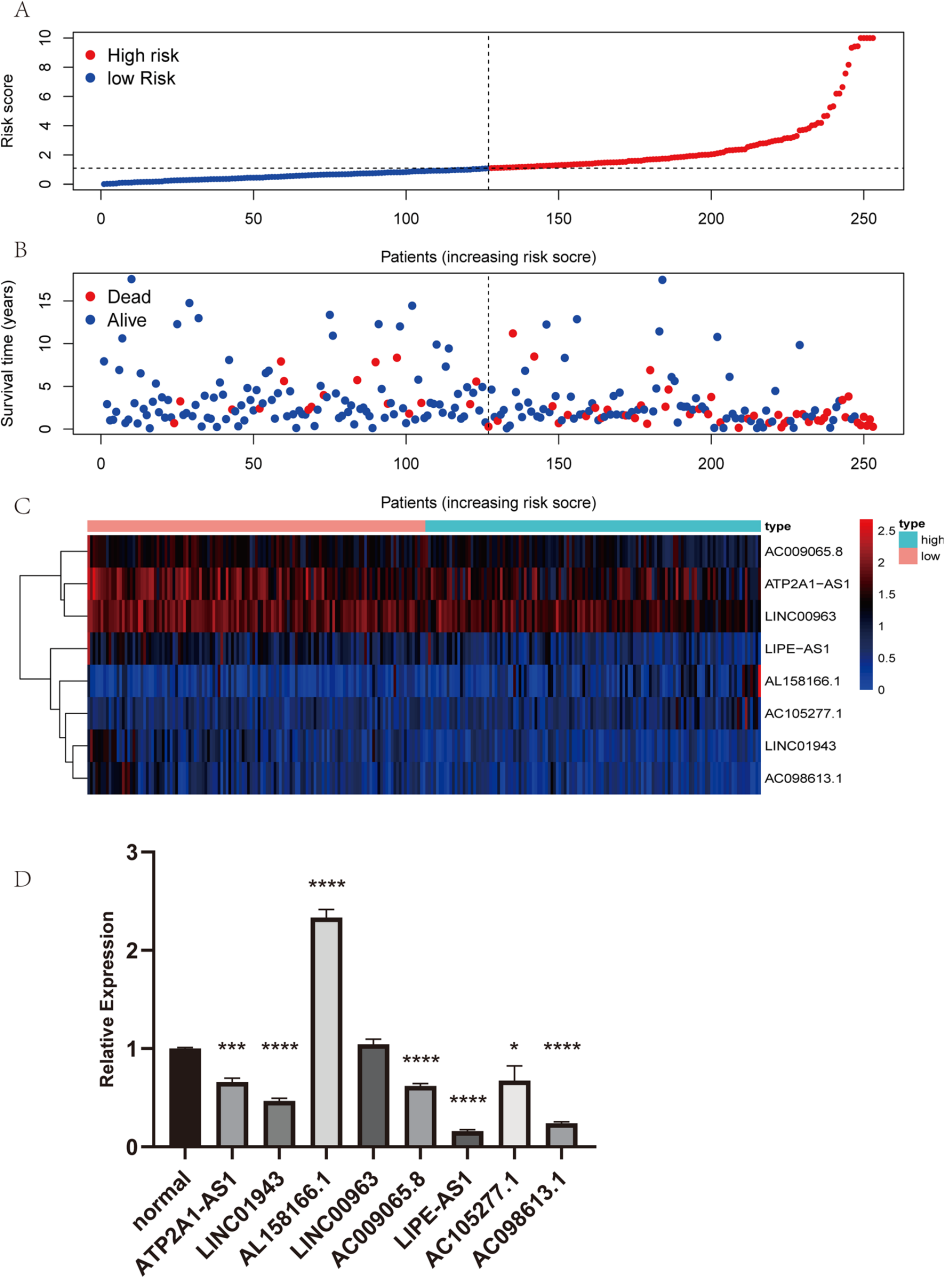
Construction of an IRlncRs-based risk score model. **A** The eight IRlncRs-based risk score distribution; **B** The eight-IRlncRs-based risk score distribution for CC patient survival status. **C** Heatmap of the eight-IRlncRs expression profiles in the high-risk and low-risk subgroups; **D** Relative expression of the 8 IRlncRs

Of the 28 IRlncRs related to the prognosis of CC (p < 0.01), 25 were low-risk factors, and 3 were high-risk factors (Additional file 3: Table S2). Eight sIRlncRs were finally incorporated to formulate the risk score model, including ATP2A1-AS1, LINC01943, AL158166.1, LINC00963, AC009065.8, LIPE-AS1, AC105277.1, AC098613.1 (Table 2). All CC samples were categorized into low-and the high-risk groups using the median risk score as a boundary (Fig. 2A). The vital status of each patient was plotted. The proportion of death events in different risk groups was also analyzed. The mortality rate increased faster in the high-risk group than in the low-risk group (Fig. 2B). The differentially expressed genes (DEGs) displayed that the expression levels of AL158166.1 and AC105277.1 had a positive coefficient and acted as risk factors. The other six sIRlncRs showed negative coefficients, including ATP2A1-AS1, LINC01943, LINC00963, AC009065.8, LIPE-AS1, AC098613.1, and served as protective factors (Fig. 2C).

**Table 2:** Eight immune-related lncRNAs identified from multivariate Cox regression analysis

Gene symbol	Ensembl ID	coef	HR	Low95	High95	*p*-value
ATP2A1-AS1	ENSG00000260442	− 0.36	0.7	0.48	1	0.05
LINC01943	ENSG00000280721	− 0.94	0.39	0.14	1.11	0.08
AL158166.1	ENSG00000227076	0.57	1.76	1.15	2.69	0.01
LINC00963	ENSG00000204054	− 0.48	0.62	0.39	0.98	0.04
AC009065.8	ENSG00000261532	− 0.56	0.57	0.3	1.07	0.08
LIPE-AS1	ENSG00000213904	− 0.58	0.56	0.29	1.09	0.09
AC105277.1	ENSG00000232453.7	0.83	2.29	1.36	3.85	0
AC098613.1	ENSG00000121797	− 0.85	0.43	0.14	1.3	0.14

To verify the clinical value of the selected sIRlncRs in predicting prognosis, we compared the expression levels of the eight sIRlncRs in cervical cancer cells to that in normal human cervical epithelial cells. As illustrated in Fig. 2D, five of the six sIRlncRs serving as protective factors showed a significant decrease in the cervical cancer cells. One of the two IRlncRs acting as risk factors showed a significant increase in the cervical cancer cells.

**Fig. 3:**
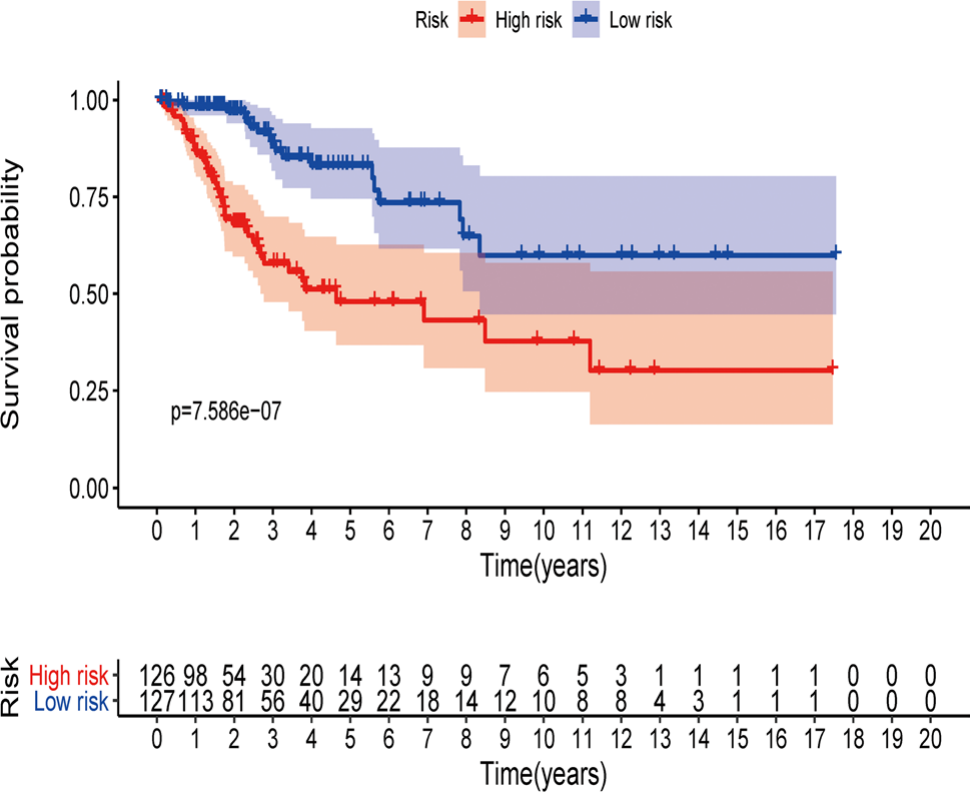
Survival curve of CC patients. Kaplan–Meier survival curve of OS among CC patients from the low-risk groups and high-risk groups. The high-risk group show the poorer prognosis

Moreover, Kaplan–Meier survival analysis was used to evaluate the above prognosis model's impact on CC patients’ survival. Survival was inferior in the high-risk group than in the low-risk group (Fig. 3).

### Independent prognostic analysis

**Fig. 4:**
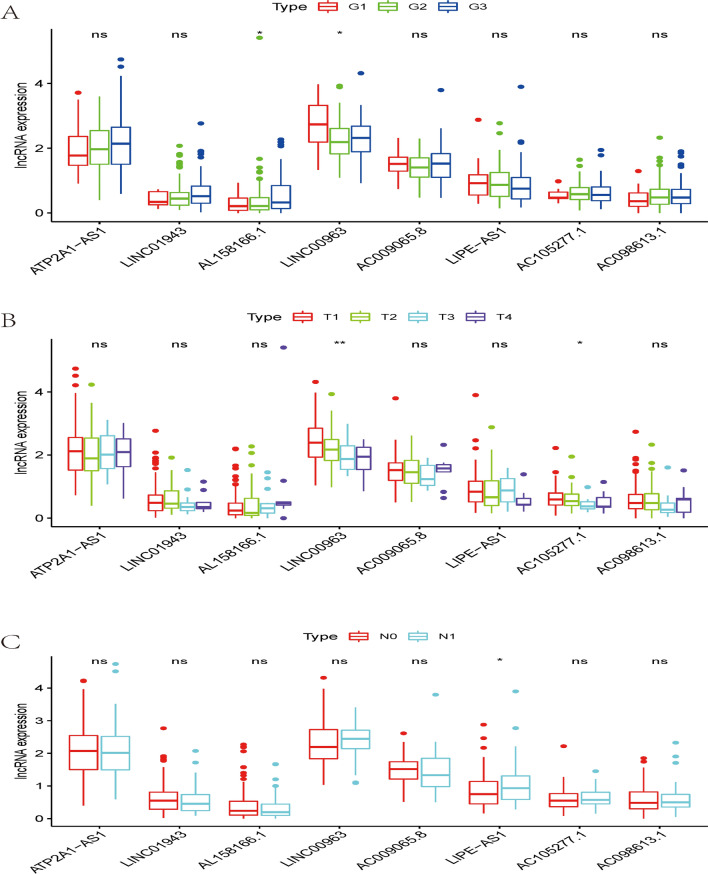
The relationships between the sIRlncRs and clinical features. **A** grading; **B** T-stage; **C** N-stage

**Fig. 5:**
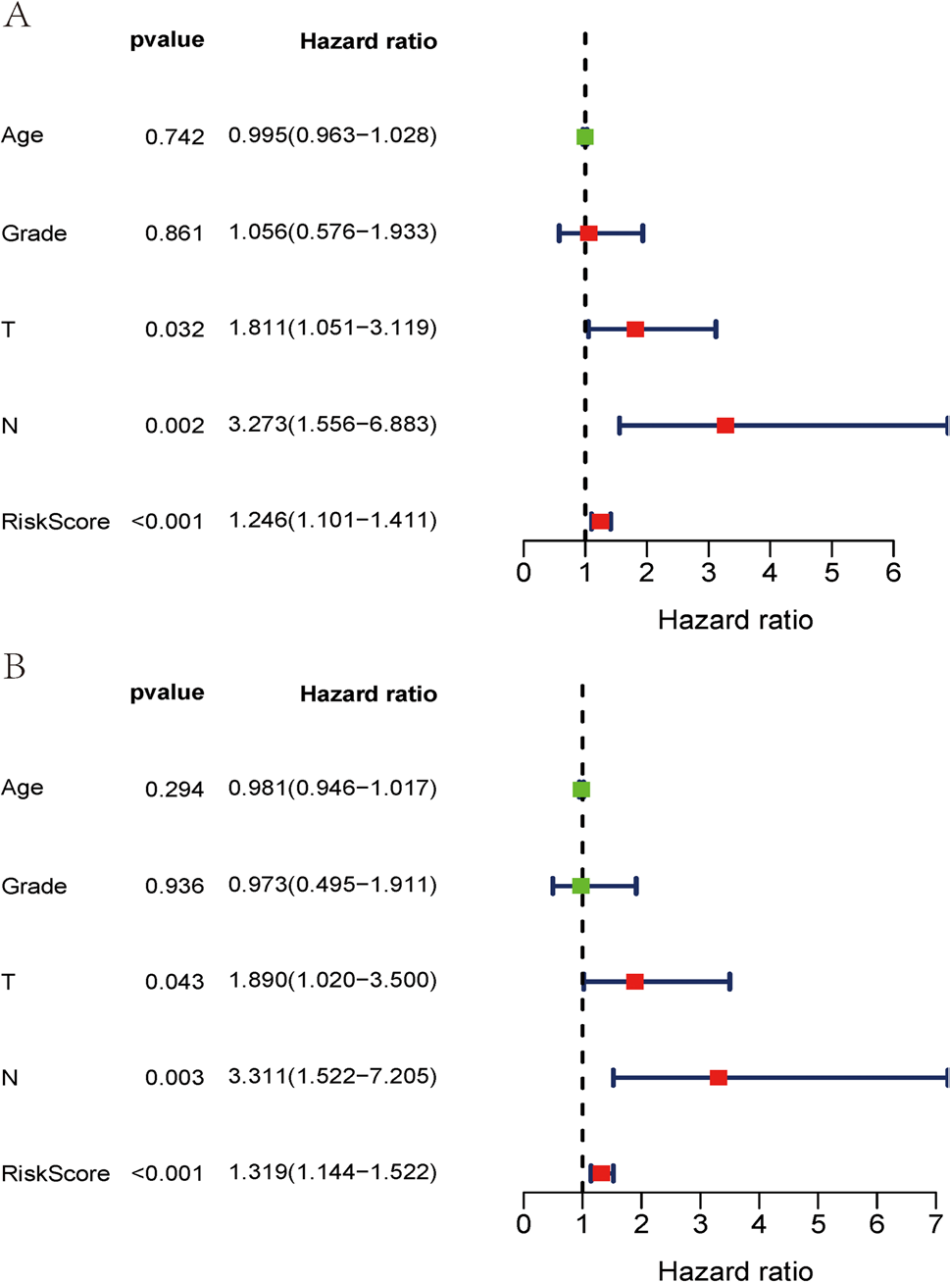
Cox regression. **A** Univariate Cox regression showed that the T stage, N stage, and risk score model were correlated with the prognosis of CC patients. **B** Multivariate Cox regression showed that the T stage, N stage, and risk score model were an independent risk factor for CC patients

**Fig. 6:**
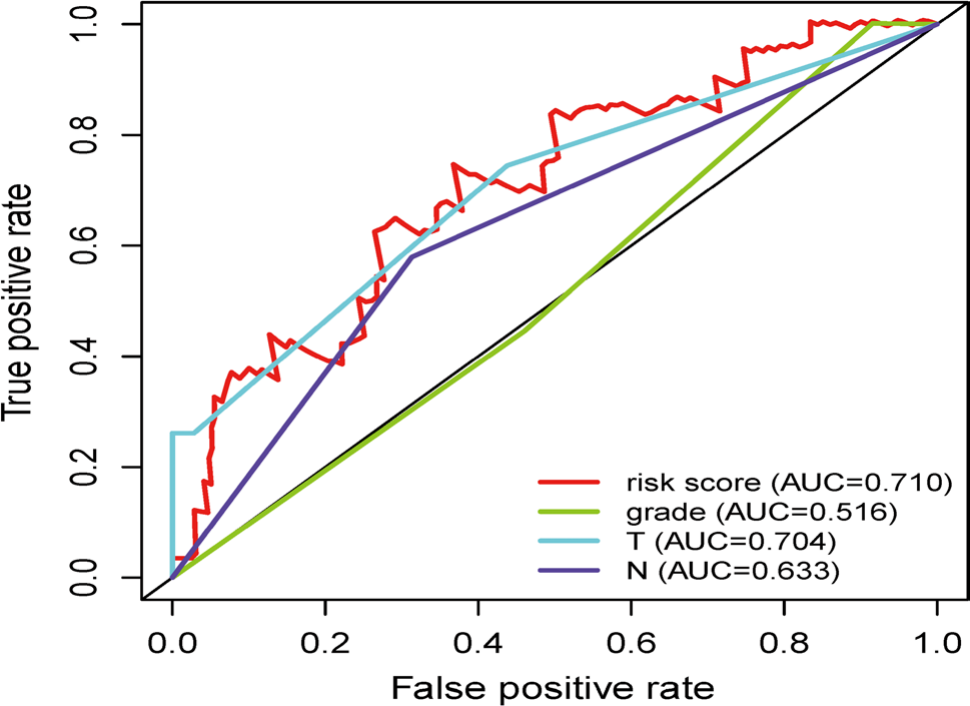
Receiver operating characteristic (ROC) curve. ROC curves demonstrated the prognostic value of the independent prognostic factors

To explore the relationship between the selected IRlncRs and clinical features of CC, the potential association of the eight IRlncRs with the clinicopathological features, including T-stage, N-stage, and tumor grading, was investigated. The results presented that the expression level of LINC00963 negatively correlated with the grading, while AL158166.1 was positively related to advanced grading (Fig. 4A). The expression of LINC00963 and AC105277.1 decreased with progressive T-stages (Fig. 4B), and the expression of LIPE−AS1 increased with the progression of the N-stage (Fig. 4C). We then performed independent risk analysis, and it showed the risk score model, N-stage, and T-stage were negatively related to the OS in univariate analysis (p < 0.05) (Fig. 5A). The results were further confirmed in the multivariate analysis showing that the risk score model, N-stage, and T-stage were significantly associated with OS (p < 0.05) (Fig. 5B). The ROC (Receiver Operating Characteristic) curve analysis validated this finding, demonstrating the predictive accuracy of the model. The AUC values for grade, T-stage, N-stage and risk score model were 0.516, 0.704, 0.633, and 0.710, respectively (Fig. 6). These results demonstrated the risk score model as an independently reliable prognostic factor.

### Construction of the nomogram

**Fig. 7:**
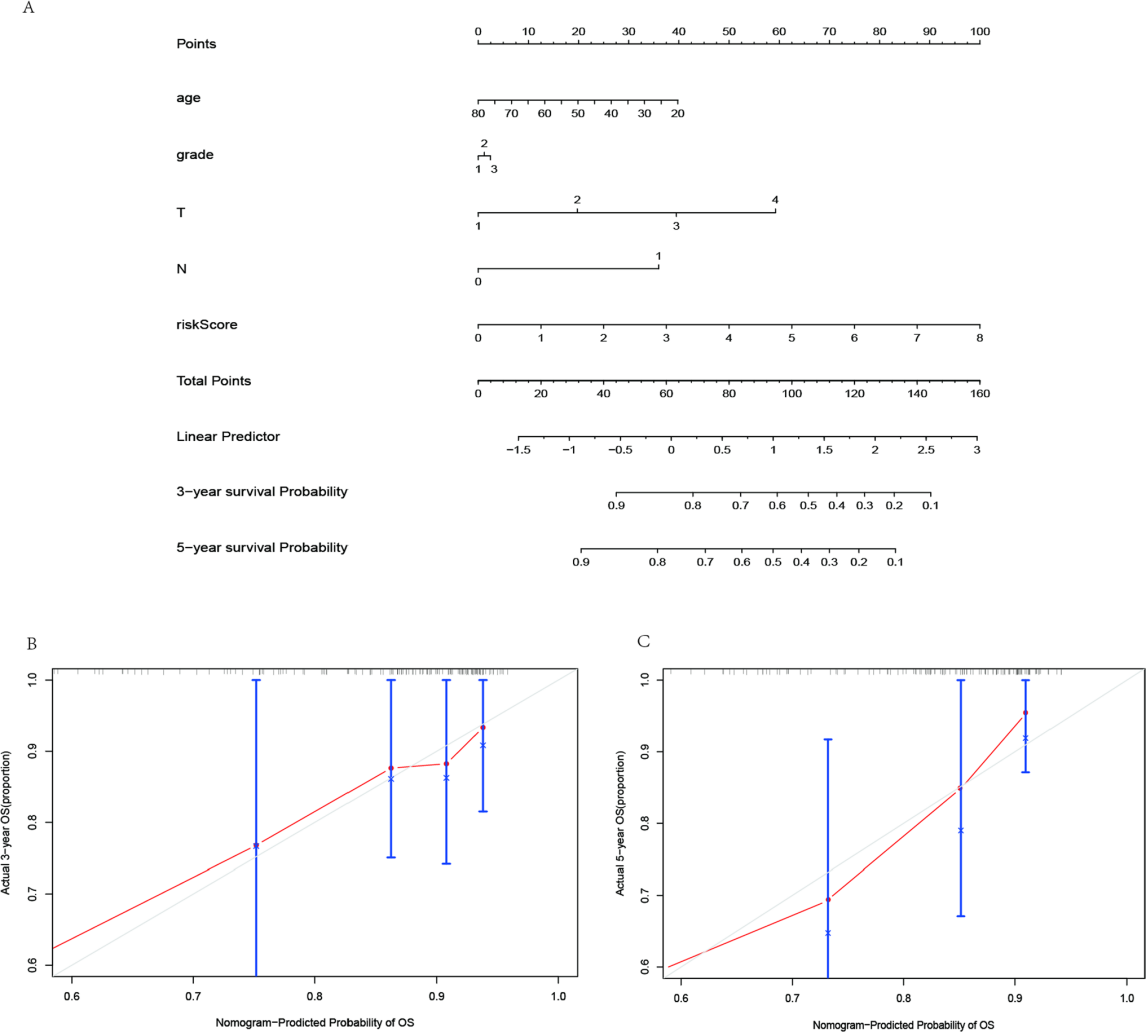
The Nomogram for predicting overall survival of CC patients. **A** The Nomogram integrating the signature risk score with the clinical characteristics for predicting OS. **B** The calibration curve for the Nomogram in TCGA cohort for predicting 3-year overall survival. **C** The calibration curve for the Nomogram in TCGA cohort for predicting 5-year overall survival

The factors of age, grade, T-stage, N-stage and risk score were further combined to construct a compound nomogram for predicting the OS of patients with CC at 3- and 5-year (Fig. 7A). The points for the factors indicated their corresponding contribution to the survival probability. The total points of each patient provided the estimated 3- and 5-year OS. The C-index of our nomogram was 0.763(95% CI 0.746–0.780, p < 0.05). The actual recurrence rate and nomogram-predicted survival rate matched well at 3 years (Fig. 7B) and 5 years (Fig. 7C), as shown by the calibration curves (Fig. 7B, C).

### The immune status of the low and high‑risk groups

**Fig. 8:**
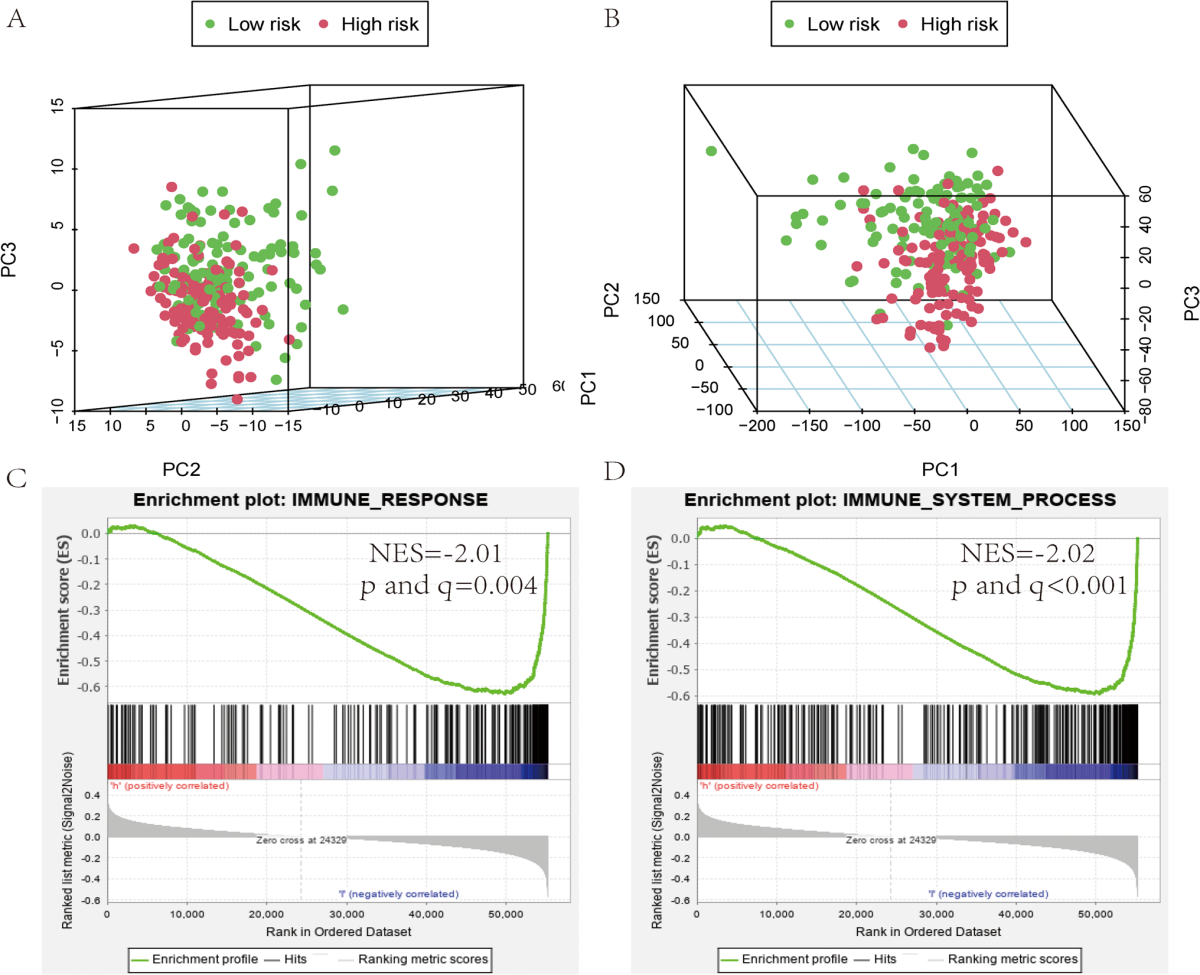
Principal components analysis (PCA) and gene set enrichment analysis (GSEA). **A** PCA plot showing high-risk group and low-risk groups based on the immune-related gene sets. **B** PCA plot showing high-risk group and low-risk group based on the whole protein-coding gene sets. **C**, **D** GSEA implied remarkable enrichment of immune-related phenotype in the low-risk group;

We performed PCA to explore the dispersion of the low-and high-risk groups based on genome-wide expression sets and the immune gene sets. Considering the immune gene sets, the low-and high-risk groups showed clustering (Fig. 8A), although there was no significant separation of the two groups based on the genome-wide expression profiles (Fig. 8B). The GSEA further verified the differences in functional annotation. As shown in Fig. 8C, D, the low-risk group's genes were predominantly mapped to the immune-related activities, such as immune response and immune system process. However, there was no gene enriched in the high-risk group (p > 0.05).

### Analysis of chemotherapeutic responses in high- and low-risk groups

**Fig. 9:**
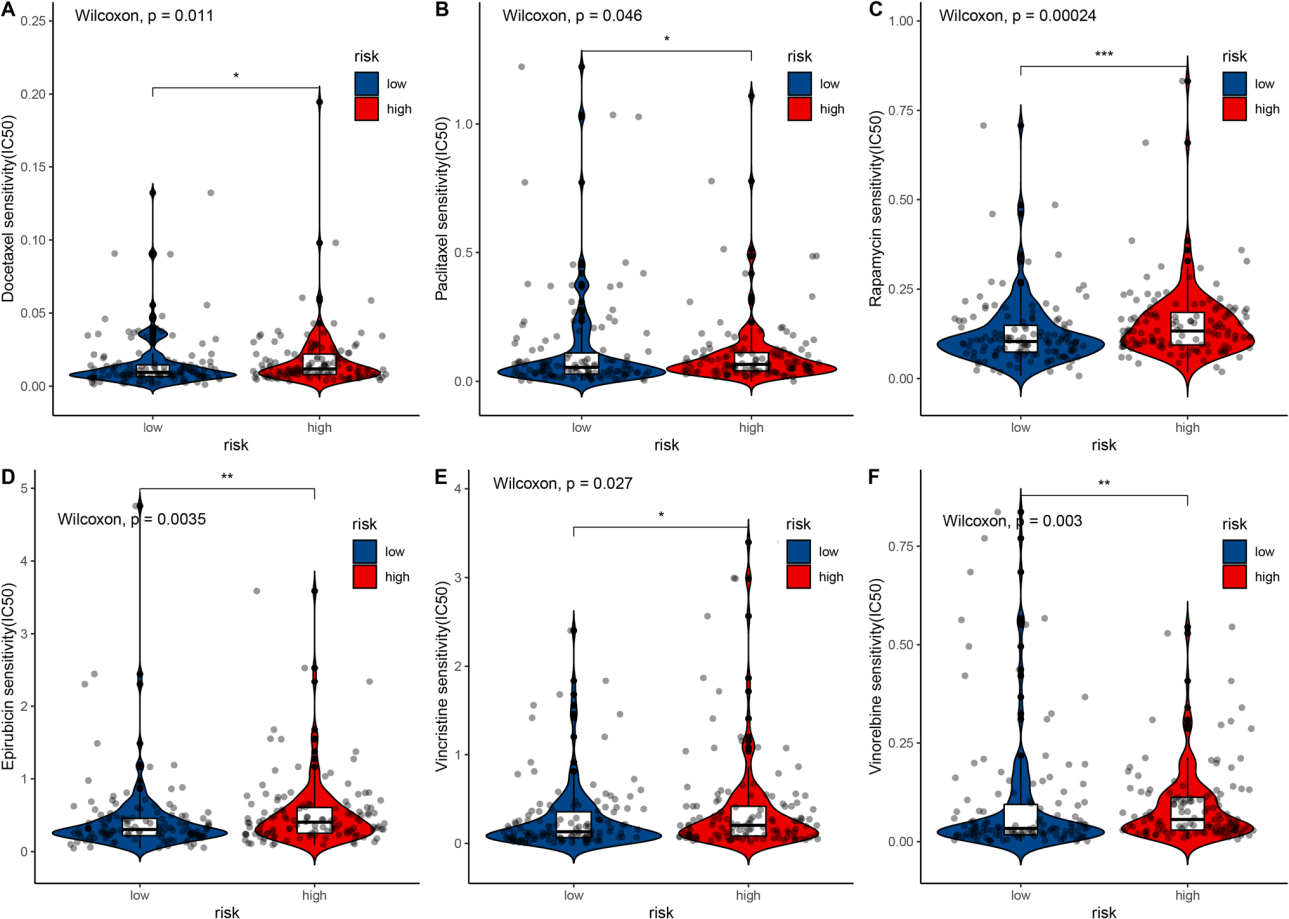
Differential chemotherapeutic responses of 6 drugs in low- and high-risk CC patients (**A**–**F**)

A total of 198 drugs were analyzed, and drug response to twenty commonly used chemotherapy drugs for CC were analyzed using the Wilcoxon rank-sum test. There were significantly lower IC50 levels for Paclitaxel, Rapamycin, Epirubicin, Vincristine, Docetaxel, and Vinorelbine in the low-risk group compared with the high-risk group (Fig. 9, p < 0.05), indicating that the low-risk group was more sensitive to these drugs. Among the 20 drugs, only docetaxel and lapatinib showed no significant difference in IC50 values (Additional file 2: Fig. S2), which indicated that our IRlncRs-based risk model might act as a potential predictor for chemosensitivity.

## Discussion

Eight IRlncRs correlated with the overall survival of CC patients were identified. The risk score model based on these eight IRlncRs demonstrated a strong ability to distinguish CC patients into low- and high-risk groups, which exhibited significant differences in OS. Further multivariate analysis showed that the eight-IRlncRs model is a valid marker of OS when accounting for other clinical characteristics, including T-stage and N-stage. The prognostic factors were further analyzed and integrated into a well-designed nomogram that demonstrated high potential for clinical application. Therefore, the eight-lncRNA model demonstrated promising value as a prognostic predictor of chemotherapeutic responses in CC. PCA based on the immune gene sets demonstrated that the low- and high-risk groups exhibited distinct immune statuses, with more abundant immune-related processes and responses observed in the low-risk group. Finally, OncoPredict analysis revealed that the tissues from the high-risk group were resistant to six commonly used chemotherapy drugs for CC.

Patients in the low-risk group may possess a better immune status, making them more sensitive to chemotherapy drugs; conversely, patients in the high-risk group might exhibit an immunosuppressed state, leading to resistance against chemotherapy drugs. The low-risk group presented enhanced antitumor immune pathways in the tumor microenvironment. Higher expression of immunostimulatory molecules promotes infiltration and activity of immune cells such as T cells and natural killer (NK) cells [19]. Robust immune activation better sensitizes tumor cells to chemotherapy through increased antigen presentation and vulnerability to immune-mediated killing [20]. In contrast, the high-risk group exhibited an immunosuppressed state. Downregulation of critical immune modulators reduces tumor immunogenicity through diminished antigen presentation and a decreased presence of cytotoxic lymphocytes [21]. This allows for immune evasion and subsequent resistance against chemotherapy, which relies on the immune system recognizing and responding to cancer cells damaged by drug treatment [22].

Certain immune cell and mRNAs predictors have been studied to predict treatment outcomes of gynecological cancer. Several risk score models based on differentially expressed genes have been developed to assess the outcomes of women with female reproductive cancers. Pan et al. reported 149 genes that were correlated with the survival of CSCC patients, and most of these genes were closely related to T cell activation [23]. Mairinger et al. developed a predictive scoring system based on immune-related genes to predict the therapy response and prognosis of epithelial ovarian cancer; however, the system was not validated for OS prediction in two datasets [24]. Yang et al. utilized 11 immune-related genes to formulate an immune signature for predicting clinical outcomes and the response to immune checkpoint inhibitors in CC patients [25]. Although several prediction models based on immune-related genes have been previously developed [23, 24], they face challenges such as a large number of genes that affect their practical utility [23] or a lack of validation across multiple datasets [24]. Compared to other mRNA-based prediction models, the lncRNA-based model constructed in this study exhibits higher specificity and provides a more precise reflection of the actual tumor condition [26]. Moreover, this study goes beyond constructing a prediction model and delves into the differences in immune status and chemotherapy sensitivity between high-risk and low-risk groups, thereby supporting the clinical application of this model. The nomogram demonstrated excellent predictive performance (C-index of 0.763), highlighting its robust potential for clinical application. Additionally, among the eight immune-related lncRNAs identified in this study, only LINC00963 and AC098613.1 have been previously reported [27, 28], while the other six have not. The current study represents the first discovery of their association with cervical cancer prognosis.

lncRNAs may have more specificity in presenting the actual tumor condition than other types of markers. A ten-lncRNA signature for predicting the survival of patients with CC showed potential value as a prognostic biomarker for CC patients (He et al. [27]). Compared to regular lncRNAs, immune-related lncRNAs (IRlncRs) are highly associated with the immune system and exhibit distinct functions in the development of tumors [29]. Regular lncRNAs primarily regulate biological processes such as tumor cell growth, proliferation, apoptosis, and migration, directly influencing tumor formation and progression. In contrast, the key role of IRlncRs lies in regulating the immune response in the tumor microenvironment and participating in the process of determining whether tumor cells can evade immune surveillance. Specifically, IRlncRs can impact antigen expression on tumor cell surfaces, alter the local immune microenvironment of tumors, and thereby affect whether tumor cells can be recognized and eliminated by immune cells. This is quite different from the direct effects of regular lncRNAs on the biological functions of tumor cells [12, 30]. Therefore, the expression levels of IRlncRs often correlate with clinical outcomes such as sensitivity to immunotherapy and prognosis [13], highlighting their advantage as tumor biomarkers.

LINC00963 participates in the progression of several types of cancers, including lung cancer [31], prostate cancer [28], and breast cancer [32]. LINC00963 can activate the oncogenic AKT/mTOR signaling pathway or EGFR signaling pathway to enhance cancer cell metastasis [28]. AC098613.1 was also included in a four-lncRNA risk score serving as an independent marker to predict the survival of bladder urothelial cancer patients [27]. However, the remaining six IRlncRs have not been reported in the literature to date, and GSEA was conducted to predict their potential functional annotations. The results showed more abundant immune-related processes in the low-risk group compared to the high-risk group. Consistent infection by human papillomavirus (HPV), the primary etiology of CC, can lead to the shutdown of host immune detection and the establishment of a local immunosuppressive status in HPV-associated CC [33]. These six IRlncRs may play a significant role in regulating these immune-related processes, and their modes of action warrant further research.

This study primarily utilized bioinformatics analysis methods to establish the association between immune-related long non-coding RNAs (IRlncRs) and the prognosis of CC, based on the reported lncRNA expression profile data from TCGA database. The use of this large-scale database significantly reduces the experimental workload and allows for efficient identification of candidate biomarkers associated with prognosis. However, relying solely on bioinformatics predictions has certain limitations, including potential biases resulting from selective population sampling, the retrospective nature that may overlook important variables, and the absence of extensive external validation to ensure wider applicability. Furthermore, the investigation of IRlncRs as prognostic markers shows promise but is still in its early stages, requiring further research to understand their complex mechanisms and interactions in CC. The insufficient comprehensive metastasis data, the necessity for more rigorous experimental validation to confirm quantitative polymerase chain reaction (qPCR) results, and the evaluation of clinical usefulness comprise the limitations and constraints of this study, providing directions for future work.

Further studies could also consider validating the accuracy of this model in peripheral blood samples. Compared to tissue samples, peripheral blood samples are more readily accessible and provide a comprehensive reflection of the body’s immune status, potentially resulting in higher accuracy of the predictive model [34]. However, the consistency of lncRNA expression patterns between tumor tissue and peripheral blood may vary depending on the cancer type and specific lncRNA. Certain lncRNAs have been explored as potential blood-based biomarkers for various cancers. For example, HOTAIR and LINC00152 show high specificity in identifying colorectal and gastric cancers, respectively. However, it is notable that the diagnostic performance of many circulatory lncRNAs is still relatively poor when detected individually, suggesting differences in their expression patterns between blood and tumor tissue [34]. Despite the challenges, developing predictive models using circulatory immune-related lncRNAs should be feasible, and this requires a multidisciplinary approach involving molecular biology, bioinformatics, clinical research, and ethical considerations.

## Conclusion

In conclusion, we identified an eight-IRlncRs signature that has the potential to be an important prognostic tool for CC patients. We expect this IRlncRs model to be practical for forecasting clinical behaviour and guide precision medicine approaches.

### Supplementary Information

**Additional file 1: Figure S1.** dot plot for the Pearson correlation test between immune-related genes and LncRNAs.

**Additional file 2: Figure S2.** Differential chemotherapeutic responses of 14 drugs in low- and high-risk CC patients.

**Additional file 3: Table S1.** Clinicopathological characteristics of CC patients from TCGA. **Table S2.** 28 immune-related lncRNAs identified from univariate Cox regression.

## Data Availability

IThe data used to support the findings of this study are available from the TCGA open database (https://tcgadata.nci.nih.gov/tcga/; LUAD).

## Abbreviations

CC: Cervical cancer
CSCC: Cervical squamous cell carcinoma
FIGO: International Federation of Gynaecology and Obstetrics
HPV: Human papillomavirus
IRlncRs: Immune-related long noncoding RNAs
lncRNAs: Long noncoding RNAs
ncRNAs: Noncoding RNAs
OS: Overall survival
sIRlncRs: Survival-related IRlncRNAs
TCGA: The Cancer Genome Atlas
TMM: Trimmed mean of M-values
PCA: Principal components analysis
GSEA: Gene set enrichment analysis
ROC: Receiver operating characteristic
AUC: Area under the curve
IC50: Half-maximal inhibitory concentration
