# Supplementary figures and images for: Immune-related LncRNAs scores predicts chemotherapeutic responses and prognosis in cervical cancer patients
Source: Discov Oncol. 2024 Apr 14;15:119. doi: 10.1007/s12672-024-00979-1 (PMC11016529; doi:10.1007/s12672-024-00979-1)

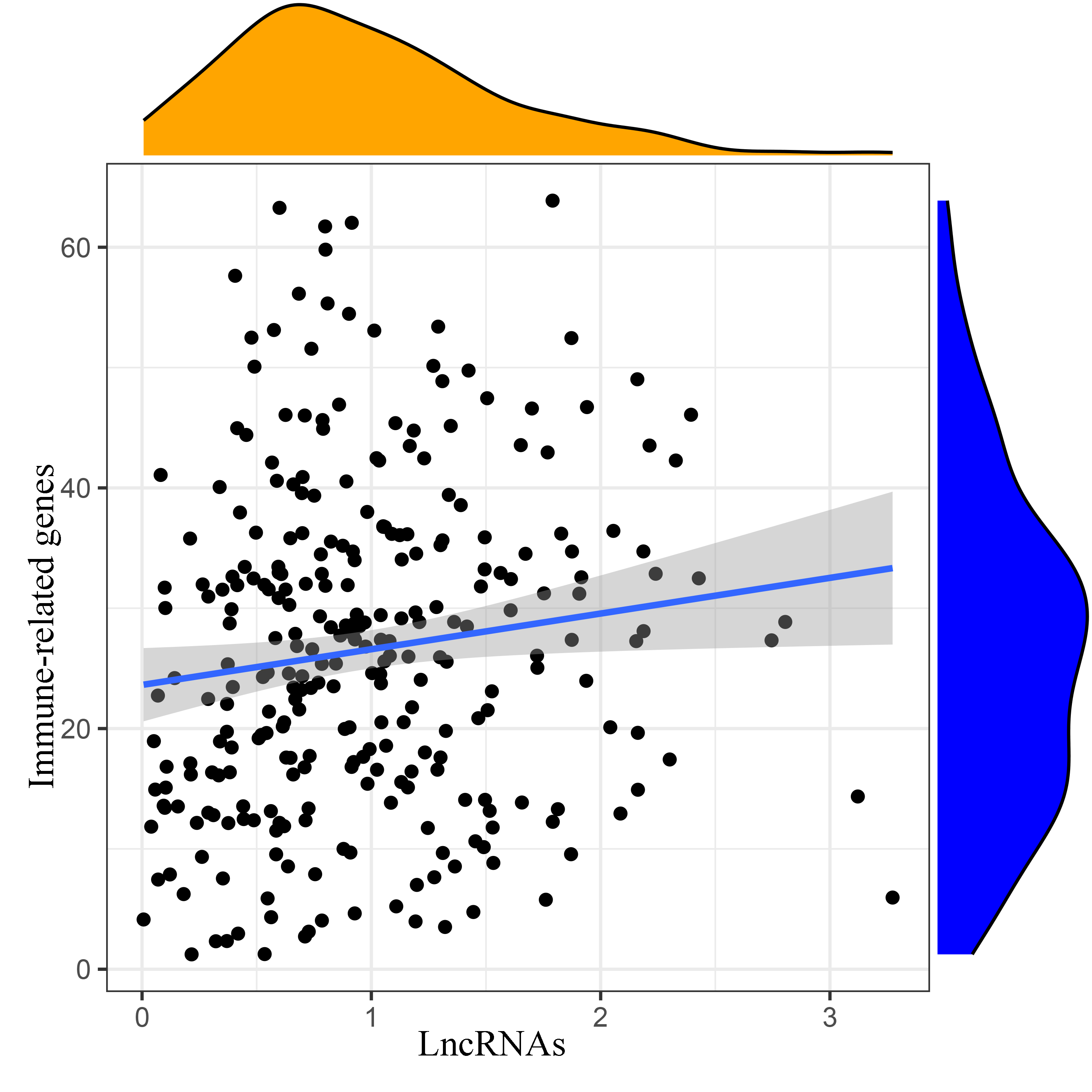

Supplement: Supplementary file 1 — Additional file 1: Figure S1. dot plot for the Pearson correlation test between immune-related genes and LncRNAs. [file 12672_2024_979_MOESM1_ESM.tif]

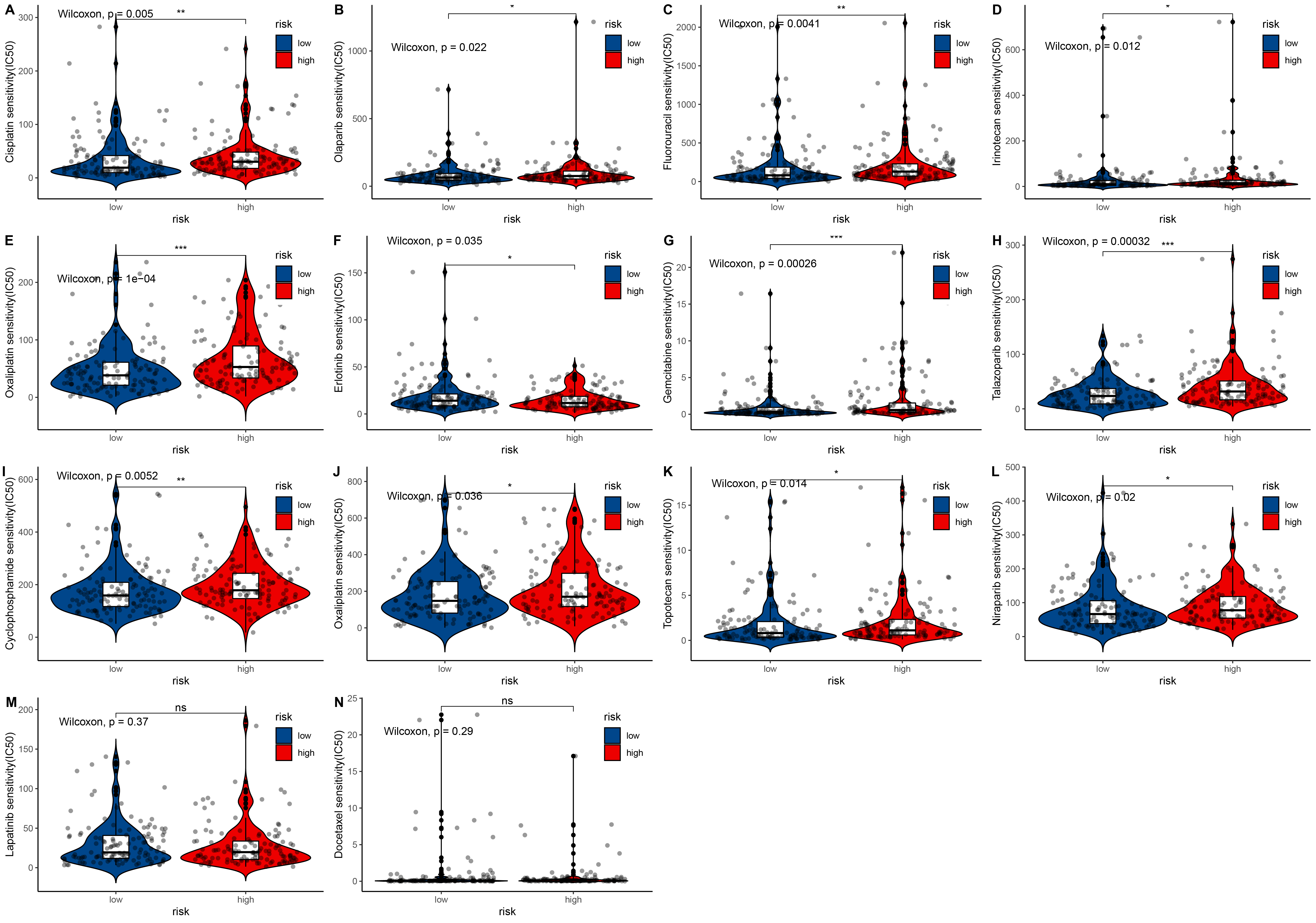

Supplement: Supplementary file 2 — Additional file 2: Figure S2. Differential chemotherapeutic responses of 14 drugs in low- and high-risk CC patients. [file 12672_2024_979_MOESM2_ESM.tif]
