# Supplementary material for: Immune-related LncRNAs scores predicts chemotherapeutic responses and prognosis in cervical cancer patients
Source: Discov Oncol. 2024 Apr 14;15:119. doi: 10.1007/s12672-024-00979-1 (PMC11016529; doi:10.1007/s12672-024-00979-1)
Supplement: Supplementary file 3 — Additional file 3: Table S1. Clinicopathological characteristics of CC patients from TCGA. Table S2. 28 immune-related lncRNAs identified from univariate Cox regression. [file 12672_2024_979_MOESM3_ESM.docx]

Table 1. Clinicopathological characteristics of CC patients from TCGA.

| Clinical characteristics |  | Total  (286) | % |
| --- | --- | --- | --- |
| Age at diagnosis (y)  Race  Grade  T classification  N classification | young age (<50)  old age (>=50)  Caucasian  African American  Asian  Unknow  Ⅰ-Ⅱ  Ⅲ-Ⅳ  Unknow  T1  T2  T3  T4  Unknow  N0  N1  Unknow | 170  116  102  88  53  43  141  115  30  131  65  20  9  61  125  57  104 | 40.56  59.44  35.66  30.76  18.53  15.03  49.30  40.21  10.49  45.8  22.73  6.99  3.15  21.33  43.71  19.93  36.36 |

Table 2: 28 immune-related lncRNAs identified from univariate Cox regression.

| Gene symbol | Ensembl ID | HR | Low95 | High95 | *p*-value |
| --- | --- | --- | --- | --- | --- |
| ATP2A1-AS1 | ENSG00000260442 | 0.59 | 0.41 | 0.84 | 0.00 |
| AC139530.1 | ENSG00000262049 | 0.45 | 0.25 | 0.81 | 0.01 |
| AC090948.3 | ENSG00000272529 | 0.36 | 0.18 | 0.71 | 0.00 |
| AC099343.2 | ENSG00000251878 | 0.36 | 0.17 | 0.75 | 0.01 |
| LINC01943 | ENSG00000280721 | 0.38 | 0.19 | 0.79 | 0.01 |
| AC010542.5 | ENSG00000275745 | 0.49 | 0.31 | 0.78 | 0.00 |
| LINC01857 | ENSG00000224137 | 0.43 | 0.24 | 0.80 | 0.01 |
| MAPKAPK5-AS1 | ENSG00000234608 | 0.45 | 0.26 | 0.77 | 0.00 |
| DBH-AS1 | ENSG00000225756 | 0.53 | 0.34 | 0.84 | 0.01 |
| AC133644.2 | ENSG00000284879 | 0.36 | 0.18 | 0.75 | 0.01 |
| PCED1B-AS1 | ENSG00000247774 | 0.58 | 0.41 | 0.83 | 0.00 |
| AL158166.1 | ENSG00000227076 | 1.94 | 1.31 | 2.87 | 0.00 |
| AL035461.2 | ENSG00000231961 | 0.65 | 0.48 | 0.89 | 0.01 |
| LINC00963 | ENSG00000204054 | 0.55 | 0.36 | 0.82 | 0.00 |
| AC009065.8 | ENSG00000261532 | 0.39 | 0.21 | 0.70 | 0.00 |
| USP30-AS1 | ENSG00000256262 | 0.66 | 0.50 | 0.88 | 0.01 |
| LINC02446 | ENSG00000256039 | 0.69 | 0.53 | 0.90 | 0.01 |
| GAS5-AS1 | ENSG00000270084 | 0.46 | 0.26 | 0.80 | 0.01 |
| TRG-AS1 | ENSG00000281103 | 0.39 | 0.19 | 0.77 | 0.01 |
| LIPE-AS1 | ENSG00000213904 | 0.37 | 0.20 | 0.71 | 0.00 |
| AC105277.1 | [ENSG00000232453.7](http://www.ensembl.org/Homo_sapiens/Gene/Summary?db=core;g=ENSG00000232453;r=1:58882874-58903747;t=ENST00000634763) | 2.41 | 1.37 | 4.26 | 0.00 |
| KMT2E-AS1 | ENSG00000239569 | 0.59 | 0.41 | 0.86 | 0.01 |
| AC009065.4 | ENSG00000260447 | 0.40 | 0.21 | 0.73 | 0.00 |
| LINC01871 | ENSG00000235576 | 0.76 | 0.62 | 0.93 | 0.01 |
| CH17-340M24.3 | ENSG00000261773 | 0.56 | 0.39 | 0.79 | 0.00 |
| AL365203.2 | ENSG00000273038 | 1.59 | 1.16 | 2.17 | 0.00 |
| AC098613.1 | ENSG00000121797 | 0.30 | 0.13 | 0.68 | 0.00 |
| AC108134.3 | ENSG00000262370 | 0.28 | 0.12 | 0.64 | 0.00 |
